# SOCIALIZING CARE FOR OLDER PEOPLE ACROSS THE COUNTRIES: A COMPARATIVE STUDY OF THE LOCATION OF CARE

**DOI:** 10.1093/geroni/igad104.3704

**Published:** 2023-12-21

**Authors:** Aeji Jang, Yeonjung Lee, Young Choi

**Affiliations:** Chung-Ang University, Seoul, Republic of Korea; Chung-Ang University, Seoul, Republic of Korea; Chung-Ang University, Seoul, Republic of Korea

## Abstract

As more people are likely to prefer to age in their own place, where and how the care in later life is provided become more important. This paper aims to compare the degree of socialization in care for older people among different countries, especially focusing on the location of care (i.e., home care and institutional care). We applied the Fuzzy-set Ideal Type Analysis to classify the countries depending on how the formal care is provided between home and institutional settings. Measures of socialization in home care include the national expenditure on home care and the number of formal care workers at home, whereas formal care workers and the number of beds in residential LTC facilities are indicators for socialization in institutional care. The four different types of formal care will be classified: HI where both home and institutional cares are highly socialized; Hi where home care is highly socialized but institutional care is not; hI where institutional care is highly socialized but not home care; and hi where neither are socialized. Results show that Denmark, Australia, and the Netherlands belongs to HI; Japan to Hi, New Zealand, Switzerland, and Germany to hI; and Korea and the United States to hi. Findings suggest that countries with high level of socialization of home care are likely to have a care management system for coordinating and monitoring services. This study provides implications for considering the unique cultural, institutional, and policy contexts among the countries in regards to the degree of socialization of care.

